# The Computer-Assisted Brief Intervention for Tobacco (CABIT) Program: A Pilot Study

**DOI:** 10.2196/jmir.2074

**Published:** 2012-12-03

**Authors:** Edwin D Boudreaux, Kristyna L Bedek, Nelson J Byrne, Brigitte M Baumann, Sherrill A Lord, Grant Grissom

**Affiliations:** ^1^University of Massachusetts Medical SchoolEmergency MedicineWorcester, MAUnited States; ^2^Philadelphia Veterans Affairs Medical CenterBehavioral HealthPhiladelphia, PAUnited States; ^3^The Credit Valley Hospital and Trillium Health CentreMississauga, ONCanada; ^4^University of Medicine and Dentistry of New Jersey, Robert Wood Johnson Medical School, and Cooper University HospitalDepartment of Emergency MedicineCamden, NJUnited States; ^5^Polaris Health Directions, Inc.Langhorne, PAUnited States

**Keywords:** technology, tobacco use cessation, smoking cessation, referrals

## Abstract

**Background:**

Health care providers do not routinely carry out brief counseling for tobacco cessation despite the evidence for its effectiveness. For this intervention to be routinely used, it must be brief, be convenient, require little investment of resources, require little specialized training, and be perceived as efficacious by providers. Technological advances hold much potential for addressing the barriers preventing the integration of brief interventions for tobacco cessation into the health care setting.

**Objective:**

This paper describes the development and initial evaluation of the Computer-Assisted Brief Intervention for Tobacco (CABIT) program, a web-based, multimedia tobacco intervention for use in opportunistic settings.

**Methods:**

The CABIT uses a self-administered, computerized assessment to produce personalized health care provider and patient reports, and cue a stage-matched video intervention. Respondents interested in changing their tobacco use are offered a faxed referral to a “best matched” tobacco treatment provider (ie, dynamic referral). During 2008, the CABIT program was evaluated in an emergency department, an employee assistance program, and a tobacco dependence program in New Jersey. Participants and health care providers completed semistructured interviews and satisfaction ratings of the assessment, reports, video intervention, and referrals using a 5-point scale.

**Results:**

Mean patient satisfaction scores (n = 67) for all domains ranged from 4.00 (Good) to 5.00 (Excellent; Mean = 4.48). Health care providers completed satisfaction forms for 39 patients. Of these 39 patients, 34 (87%) received tobacco resources and referrals they would not have received under standard care. Of the 45 participants offered a dynamic referral, 28 (62%) accepted.

**Conclusions:**

The CABIT program provided a user-friendly, desirable service for tobacco users and their health care providers. Further development and clinical trial testing is warranted to establish its effectiveness in promoting treatment engagement and tobacco cessation.

## Introduction

For a tobacco intervention to be implemented routinely in most health care settings—especially fast-paced settings like a hospital emergency department or a busy primary care clinic—it must be brief, convenient, and require little specialized training [1,2]. Traditionally, tobacco cessation interventions have not met these requirements, contributing to a lack of translation of empirically supported interventions into clinical care [3,4].

Computer programs have the potential to make tobacco screening and cessation more convenient, tailored to the individual patient, and uniformly applied, while taking less provider time and requiring less provider training to implement properly. Prior research supports the feasibility and effectiveness of a variety of technological components useful for tobacco assessment and intervention, including computerized assessments [5-13], video education [10,11], provider prompts [11,14-18], personally tailored feedback reports [5,6,11,12,19-22], and the option for an automated self-referral to tobacco cessation providers [5].

To our knowledge, there are currently no programs that blend these features into one integrated program that can be used in busy health care settings. For this reason, we created the Computer-Assisted Brief Intervention for Tobacco (CABIT) software, which is designed to facilitate brief tobacco cessation treatment and referrals during or immediately after a health care visit. It was designed to be used even in time-demanding settings like a hospital emergency department. This paper describes our development of the CABIT, its functionality, and our initial pilot testing and evaluation.

## Methods

### CABIT Overview

**Figure 1:**
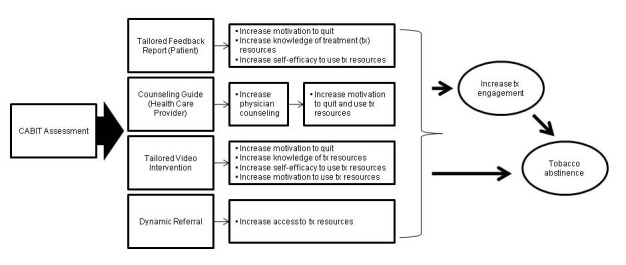
Conceptual model of the CABIT program and its mechanism of action.

Using the published literature [8,13,20-22] and our project team’s experience, we created an initial draft of the CABIT and assessed its design and functionality. During this process, we identified areas where further input from end users was needed. Then we conducted focus groups and key informant interviews with end users. Our sample was comprised of 22 health care professionals from various specialties (eg, emergency medicine, internal medicine, etc) and 13 smokers with varying levels of motivation to quit. We gathered opinions pertaining to the features that would make the CABIT (1) practical, (2) effective at improving motivation to quit smoking, and (3) useful in facilitating linkage with tobacco treatment resources. This qualitative data guided the refinement of the CABIT’s technical specifications. Figure 1 provides a conceptual model of the CABIT program and its hypothesized mechanisms of action.

The CABIT is a web-based program comprised of 4 integrated modules: (1) a computerized assessment of tobacco use and related psychosocial variables, (2) a stage-of-change-based video intervention, (3) a referral generator, and (4) a report generator. Theoretically driven, each CABIT module was informed by principles derived from Motivational Interviewing (MI) [23], the Transtheoretical Model (TTM) [24,25], the Decisional Balance Theory [26,27], and the Social Learning Theory [28]. MI was the primary treatment approach used to guide the choice of assessments utilized in the program and to design the message framing for the feedback reports [23]. MI seeks to help the patient resolve ambivalence about change. Essential to the approach is a respectful, compassionate, client-centered attitude that emphasizes autonomy and choice. The CABIT incorporates prominent MI-based principles. First, it assesses motivation for change and self-efficacy. Second, it provides tailored feedback of the assessments, including nicotine dependence, decisional balance, and self-efficacy, to help reduce ambivalence. Third, MI is the primary therapeutic approach used to design the message framing for the videos and feedback reports. The videos and reports offer nonauthoritative and nonconfrontational guidance for behavior change and encourage the health care provider to adopt this approach as well. The CABIT also stresses collaborative goal setting, using a menu of treatment options. Finally, the CABIT focuses on treatment entry. The CABIT’s development was also influenced by the TTM, which is highly compatible with MI and includes components from the Decisional Balance Theory [26,27] and the Social Learning Theory [28]. Specifically, we used measures of the stages of change, pros and cons of smoking, and self-efficacy, all of which are well-validated components of TTM. The stage-of-change assessment was the primary organizing structure used to cue the stage-matched videos, reinforce the overall “personally tailored” tone of the Patient Tobacco Feedback Report and the Health Care Provider Report, and provide specific content for the reports (ie, pro/con evaluation, high-risk situations). Altogether, each component of the CABIT is designed to directly or indirectly address a patient’s motivation to quit, motivation to use effective treatment, knowledge of treatment resources, and ability to access and use treatment resources.

### Patient Assessment

**Figure 2:**
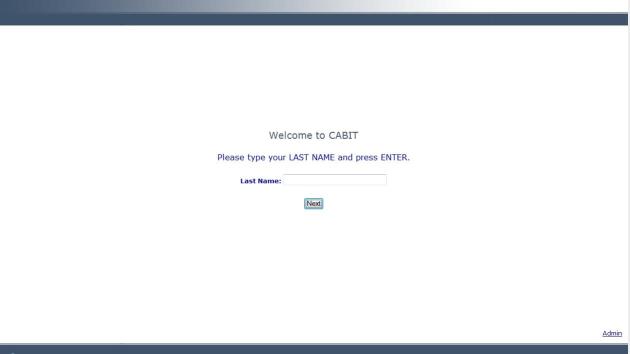
The welcome screen of the CABIT program.

**Figure 3:**
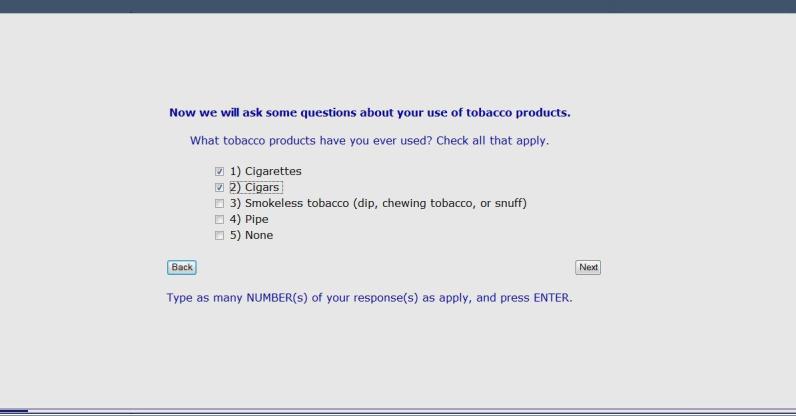
Screenshot from the CABIT program showing questions about tobacco products used.

**Figure 4:**
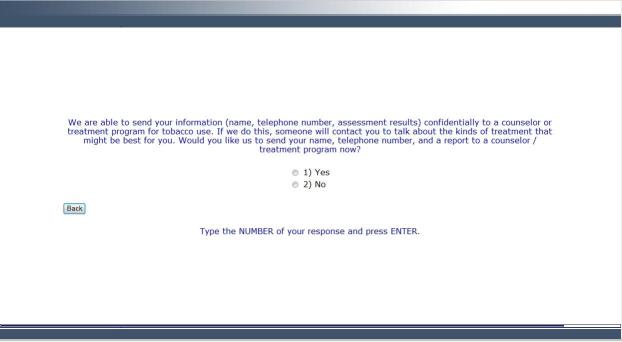
Screenshot from the CABIT program showing how patients are asked if they would like a referral to a treatment program.

**Figure 5:**
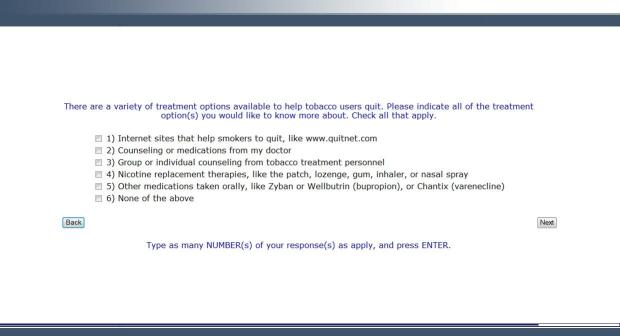
Screenshot from the CABIT program showing treatment options for patients.

The CABIT software’s response-adaptive programming logic ensures each participant is presented with questions appropriate to his or her situation and previous responses. For example, a patient is asked, “Which tobacco products have you ever used?” A patient is then only asked about current use for types of tobacco that he or she indicated ever using. Further, assessments specific to type of tobacco use were chosen and the general wording of remaining assessment items were tailored to type of tobacco use. Based on a commonly used staging algorithm [24,25], each individual is assigned a stage of change: precontemplation, contemplation, preparation, or action. Patients in the precontemplation stage are current tobacco users not intending to quit or intending to quit over 6 months in the future. Those in the contemplation stage are current tobacco users intending to quit in the next 6 months. Patients in the preparation stage are current tobacco users intending to quit in the next 30 days. Those in the action stage quit using tobacco for longer than 1 day less than 6 months ago. These stages of change serve as the cue for the stage-matched videos and guide the overall tone and content of the counseling guide for the health care provider (Health Care Provider Report) and the tailored feedback report for the patient (Patient Tobacco Feedback Report). Information obtained from the assessment, including patient-selected items from a menu of treatment options and tobacco educational resources, is integrated into the tailored Patient Tobacco Feedback Report. Patients indicating interest in quitting or staying quit are offered an automated faxed referral to a local tobacco treatment provider (ie, a dynamic referral, further discussed in the Referral Generator section). More detail regarding the CABIT assessment is included in the Measures section below. Figures 2-5 provide examples of screenshots from the program.

### Video Intervention

Upon completion of the assessment, the CABIT cues the appropriate stage-matched tobacco education video. Three already available educational videos tailored to 4 stages of change (precontemplation, contemplation, preparation, and action) were used. Although not ideal from a tailoring perspective, patients in both the contemplation and preparation stages viewed the same video. This 6-7 minute video was professionally produced and used appealing graphics to reinforce and illustrate important points related to tobacco use and cessation. For example, videos provided information about the stages one progresses through when getting ready to quit, consequences of tobacco use, benefits of quitting, and tips on quitting and available resources.

### Referral Generator

The referral generator uses technology developed in a previous study called the Dynamic Assessment and Referral System for Substance Abuse (DARSSA) [5]. In addition to receiving a printed list of tobacco dependence treatment programs tailored to the individual’s geographic location and insurance type (ie, passive referral), patients considering quitting are given the option of an automated faxed referral (ie, dynamic referral). If the participant agrees to release his personal contact information, the CABIT faxes the referral to the “best matched” provider in the referral library based on the patient’s zip code and insurance status. The tobacco dependence programs in the referral library agreed to contact the patient within 5 days of receiving a dynamic referral to complete an initial phone screening, discuss treatment options, and, if interested and appropriate, schedule an intake assessment.

### Report Generator

The report generator produces 3 reports based on information the patient provided through the CABIT assessment: (1) Patient Tobacco Feedback Report, (2) Health Care Provider Report, and (3) Tobacco Treatment Referral (generated only for participants who choose a dynamic referral).

#### Patient Tobacco Feedback Report

The patient received a personally tailored report written at an eighth-grade reading level. It was crafted using principles of Motivational Interviewing [23] and gain-frame (versus loss-frame) messaging strategies [29]. The length of the feedback report varies based on participant’s assessment responses and information requested. The report includes a referral summary, which lists tobacco treatment resources the patient may contact and information about where the dynamic referral was sent, if it was chosen. The report also includes a personalized summary with feedback on the participant’s tobacco use history, stage of change, readiness to quit, benefits of quitting, money spent on tobacco, level of addiction, temptations or triggers to using tobacco, and perceived risks of quitting. Additionally, participants are provided with information about resources for quitting and other tobacco-related topics that he or she selected when completing the assessment. See Multimedia Appendix 1 for an example of a Patient Tobacco Feedback Report.

#### Health Care Provider Report

The one-page counseling guide for the health care provider summarizes the tobacco use information that we deemed most important for clinical decision making based on our focus groups and in-depth interviews. It uses responses patients provided in the CABIT to provide evidence-based guidance for counseling the patient based on the NCI’s Five As (Ask, Advise, Assess, Assist, Arrange follow-up) [3]. The Ask/Assess section provides a summary of the patient’s assessment, including the patient’s smoking history, perceived symptoms or illnesses related to tobacco use, readiness to quit, factors related to poor outcomes (eg, living with a smoker, depression), and interest in assistance from provider. The Advise/Assist section provides counseling guidance for the provider, including presenting a clear but nonjudgmental recommendation that the patient consider quitting tobacco use and stage-based suggestions to help facilitate quitting. The Refer/Arrange section includes the list of resources provided to the patient and where the dynamic referral was sent if the patient opted to receive one. See Multimedia Appendix 2 for an example of a Health Care Provider Report.

#### Tobacco Treatment Referral

This report, faxed to the “best matched” provider for patients who choose a dynamic referral, provides patient contact information and a summary of the patient's assessment. In particular, this report provides information on the patient’s tobacco use, level of addiction, tobacco-related illnesses or symptoms, past attempts to quit, methods used to quit, and readiness to quit. It also included personal factors related to poor prognosis and the patient’s readiness to quit ruler. See Multimedia Appendix 3 for an example of a Tobacco Treatment Referral.

### Setting and Population

The CABIT program was implemented in three settings in a large hospital system in New Jersey (Cooper University Hospital): the Emergency Department; the Employee Assistance Program serving employees of the hospital system; and the outpatient Tobacco Dependence Program associated with the hospital. The Emergency Department is an academic, urban, Level I trauma center serving a catchment area of approximately 2 million people. The annual census is approximately 47 000 visitors, 20% of whom are admitted to the hospital. The Emergency Department and Employee Assistance Program demonstrate the CABIT’s utility across environments with different paces, procedures, staffing, patient characteristics, and foci of care. The Tobacco Dependence Program, with its specialized focus on tobacco treatment, yielded a cohort of smokers, recent quitters, and tobacco treatment counselors who were able to provide topical advice on the program.

### Participant Selection

The recruitment protocol in the Emergency Department was similar to our published studies [30-32]. Research assistants approached adult patients at their bedside after they had been clinically evaluated and stabilized. In the Employee Assistance Program, participants were recruited with a system-wide email under the auspices of the program announcing a new computerized tobacco cessation program for employees. Interested employees were directed to contact the research staff. In the Tobacco Dependence Program, participants were recruited by counselors and those interested were referred to the research assistants. Adults in these three settings who were current tobacco users or who recently quit (in the past 6 months), who could read and understand English, who could read words on a computer screen, and who did not meet exclusion criteria were invited to participate in the study. Exclusion criteria included being under 18 years of age, being a nonsmoker or having quit over 6 months ago, having severe illness or distress (eg, intubation, severe pain, vomiting), having cognitive insufficiency (eg, dementia, psychosis, altered consciousness), having insurmountable language barriers (eg, non-English speaking), and refusing to participate. Participants were reassured that neither prior computer experience nor a desire to quit was required in order to participate.

### Procedure

After the prototype of the CABIT finished laboratory testing, we completed a pilot test with 20 patients recruited from the Emergency Department. This pilot test was designed to assess global functionality, gain experience with the CABIT in a clinical setting, and reconcile problems with the software. Following resolution of problems, the CABIT was fully administered with updated components in the Emergency Department, the Employee Assistance Program, and the Tobacco Dependence Program during 2008. This was referred to as the Field Evaluation Study because the intent was to assess how feasible it was to fully integrate the CABIT into these clinical field settings. This research was approved by the institutional review boards for Cooper University Hospital and Polaris Health Directions, Inc.

Patients were verbally asked to participate in the study if they agreed to answer screening questions and were eligible to participate based on their responses. This involved describing the study and the risks and benefits of participating to potential participants, and advising participants that they may withdraw from the study at any point in time. Written consent was obtained from all participants who verbally agreed to participate.

The assessment was self-administered and research assistants were available to answer questions and to solve problems, if needed. Following completion of the patient assessment and viewing of the stage-matched video, research assistants reviewed feedback reports with the patients. Research assistants then conducted a satisfaction assessment following completion of the CABIT program to obtain impressions from participants in all settings and from the participant’s health care provider (ie, physician, nurse, or counselor) in the Emergency Department and Tobacco Dependence Program. Since participants from the Employee Assistance Program were recruited directly, they essentially did not have a provider to evaluate the program.

To gather more detailed evaluations, 15 of the 67 pilot test participants completed an in-depth interview pertaining to a particular component of the CABIT program (assessment, *n* = 5; video intervention, *n* = 5; tailored patient feedback report, *n* = 5). After patients completed the program, they were asked the satisfaction assessment questions and additional open-ended questions about the randomly assigned CABIT components. These interviews were recorded for later review and analysis for themes.

### Four-Week Follow-Up

Research assistants contacted Field Evaluation Study participants 4 weeks after they completed the CABIT program to determine treatment initiation and to re-assess tobacco use. Subjects recruited from the Tobacco Dependence Program were not followed because they were already in tobacco treatment. For participants who chose a dynamic referral, a research assistant contacted the tobacco treatment provider 4-8 weeks after the participant completed the CABIT program to verify the patient’s report of entering treatment.

### Measures

#### CABIT Assessment

Because of the pilot nature of the study, we included a broad range of well-established instruments that are robustly associated with tobacco abstinence and rooted in the theoretical traditions listed in the CABIT Overview section. Table 1 provides a description of the measures and the references.

**Table 1:** Assessment measures used or adapted for the CABIT.

Assessment Measure	Construct	Source
Behavioral Risk Factor Surveillance System (BRFSS) survey questionnaire	Tobacco use	Centers for Disease Control and Prevention (CDC), 2006 [33]
Fagerström Test of Nicotine Dependence (FTND)	Level of nicotine addiction	Heatherton et al., 1991 [34]
Fagerström Test of Nicotine Dependence—Smokeless Tobacco (FTND-ST)	Level of nicotine addiction for smokeless tobacco	Ebbert et al., 2006 [35]
Smoking: Stages of Change (short form)	Stage of change	DiClemente et al., 1991 [36]; Velicer et al., 1995 [37]
Readiness Rulers	Importance, readiness, and commitment to tobacco cessation	Biener and Abrams, 1991 [38]
Perceived Risks and Benefits Questionnaire (PRBQ)	Perceived risks and benefits associated with tobacco cessation	McKee et al., 2005 [39]
Reasons for Quitting (RFQ)	Reasons for tobacco cessation	Curry et al., 1990 [40]
Wisconsin Inventory of Smoking Dependence Motives (WISDM-68)	Motivation for tobacco use	Piper et al., 2004 [41]
Smoking: Self-Efficacy for Smoking/Temptation (short form)	Self-efficacy for smoking cessation and temptations for smoking	Velicer et al., 1990 [42]
Smoking Consequences Questionnaire (SCQ)	Smoking outcome expectancies	Brandon and Baker, 1991 [43]
Decisional Balance for Smoking (short form)	Pros and cons of smoking	Velicer et al., 1985 [44]
Perceived Health Risks	Perceived health risk of tobacco use	Bock et al., 2001 [45]
Perceived Risks	Perceived risks of tobacco use	Hampson et al., 2000 [46]
Patient Health Questionnaire-2 (PHQ-2)	Two-item depression screener	Kroenke et al., 2003 [47]

#### Satisfaction Assessment

##### Patient Satisfaction Assessment

The satisfaction assessment for patients consisted of semi-structured interviews assessing impressions of the CABIT assessment program, reports, and referrals, along with quantitative ratings. Suggestions for improving the CABIT were also elicited. Quantitative ratings were obtained for domains using a 5-point scale (1 = Very Poor; 2 = Poor; 3 = Fair/Average; 4 = Good; 5 = Excellent). Domains assessed with participants included those related to the different components of the CABIT. For the assessment domain, participants were asked about clarity of instructions, ability to read words on the computer screen, ease of responding to questions using the keyboard, understandability of how to return to the previous question, length, comfort in answering honestly, and appropriateness of questions. For the video, participants were asked about length, understandability and usefulness of information presented, ability to maintaining interest, and effectiveness in changing attitude regarding tobacco use. For the Patient Tobacco Feedback Report, participants were asked about understandability and usefulness of information, effectiveness in changing attitude regarding tobacco use, and usefulness of resources. These domains were patterned after published work on the Dynamic Assessment and Referral System for Substance Abuse (DARSSA) [5].

##### Patient Satisfaction Assessment with Depth Interview

In addition to the quantitative ratings, participants who completed the depth interviews were asked open-ended questions about a randomly assigned CABIT component (assessment, video intervention, or tailored feedback report). This included questions to help participants further elaborate their feedback on the domains from the satisfaction assessment, overall impressions of the program, and allow for suggestions for improvement.

##### Health Care Provider Satisfaction

For participants enrolled in the pilot test through the Emergency Department or Tobacco Dependence Program, the participant’s treating physician, nurse, or counselor provided satisfaction ratings of the CABIT process and the Health Care Provider Report, including: understandability and usefulness of information, length, overall format, provision of information not assessed, effects on how provider would manage the patient, and whether the patient would have received a referral if he or she did not participate in the CABIT program.

### Statistical Analysis

Nonparametric summary statistics, including means and standard deviations, were calculated for all variables, including the end-user satisfaction ratings, completion time for the assessment, and 4-week outcomes. *A priori,* we choose a target mean satisfaction rating of ≥ 4.00 on the 5-point scale for each domain assessed by the end user. The domains failing to meet this goal would need to be modified and reassessed prior to the Phase II efficacy trial. Since the present study is a proof-of-concept study designed to assess the functioning, usefulness, and acceptance of the CABIT, treatment initiation and abstinence were considered secondary outcomes.

## Results

### Descriptive Characteristics

**Figure 6:**
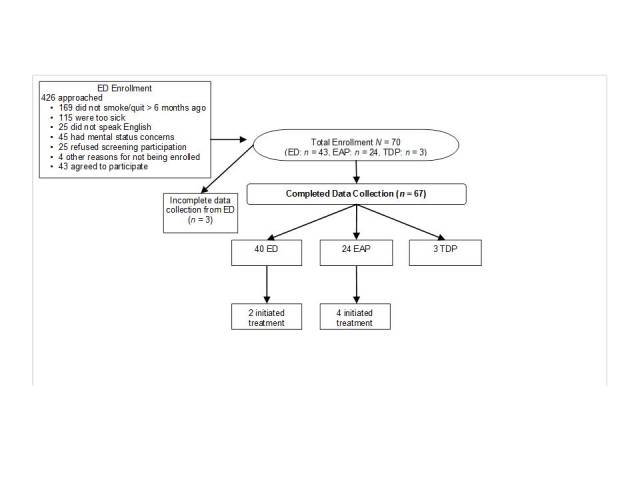
Enrollment of participants in the Field Evaluation Study of the CABIT program. Participants were recruited from the Emergency Department (ED), Employee Assistance Program (EAP), and Tobacco Dependence Program (TDP). Screening data was only available for participants from the emergency department. Follow-up was not completed with patients in the tobacco dependence program as they were already receiving treatment. Treatment initiation was confirmed through contact with tobacco dependence specialists in the referral library.

For the Field Evaluation Study, 426 patients were approached for participation in the Emergency Department. Of these patients, 169 did not smoke, 115 were too sick, 25 did not speak English, there was concern about mental status for 45 patients, 25 patients refused to be screened or participate, and 4 had other reasons for not participating. A total of 43 patients were enrolled during the Field Evaluation Study in the Emergency Department, but 3 failed to complete the program. Twenty-four participants were enrolled in the Employee Assistance Program and 3 from the Tobacco Dependence Program. Information about the number of patients invited to participate in the Employee Assistance Program was not recorded as hospital employees contacted research staff directly. Similarly, research staff were provided with contact information for interested patients in the Tobacco Dependence Program, so the number of patients invited to participate from this program was not recorded. A total of 67 participants completed the CABIT assessment across all settings (Emergency Department, *n* = 40; Employee Assistance Program, *n* = 24; and Tobacco Dependence Program, *n* = 3) (see Figure 6). Reasons for not completing the assessment included patients being discharged or being taken for a procedure or testing. Table 2 summarizes the participants’ characteristics.

**Table 2:** Demographic and smoking characteristics of participants who completed the CABIT program (n = 67).

Characteristic			Data
**Age**			*M* = 42 (*SD* = 12.69)
**Gender**			
	Male		21 (31%)
	Female		46 (69%)
**Marital status**			
	Never married		23 (34%)
	Married or remarried		22 (33%)
	Divorced or separated		9 (13%)
	Other marital status		13 (19%)
**Race/ethnicity**			
	Caucasian		35 (52%)
	African-American		21 (31%)
	Hispanic only		6 (9%)
	White Hispanic		1 (2%)
	Black Hispanic		0 (0%)
	Other		4 (6%)
**Education level**			
	8th grade education or less		0 (0%)
	Some high school		13 (19%)
	High school graduate		24 (36%)
	Some college		20 (30%)
	College graduate		9 (13%)
	Some graduate work		1 (2%)
**Average years of tobacco use**			*M* = 26.22 (*SD* = 11.98)
**Current tobacco use (some or every day)**			
	Cigarettes		58 (87%)
	Cigars		9 (13%)
	Pipe		1 (1%)
	Smokeless tobacco		0 (0%)
**Daily amount of tobacco use by type for tobacco of choice ^a^**			
	**Cigarettes**		(*n* = 57)
		1-10 per day	26 (46%)
		11-20 per day	20 (35%)
		21-30 per day	11 (19%)
	**Cigars**		(*n* = 4)
		2-3 per day	2 (50%)
		4-5 per day	1 (25%)
		6 or more	1 (25%)
**Stage of change**			
	Precontemplation		17 (25%)
	Contemplation		25 (37%)
	Preparation		20 (30%)
	Action		5 (8%)
**Positive on depression screen**			17 (25%)
**Positive on risky alcohol screen**			27 (40%)
**Positive on drug use screen**			16 (24%)

^a^ Data was not available for pipe and smokeless tobacco use as participants did not indicate that these products were the most frequently used.

### Satisfaction

#### Patient Satisfaction

**Figure 7:**
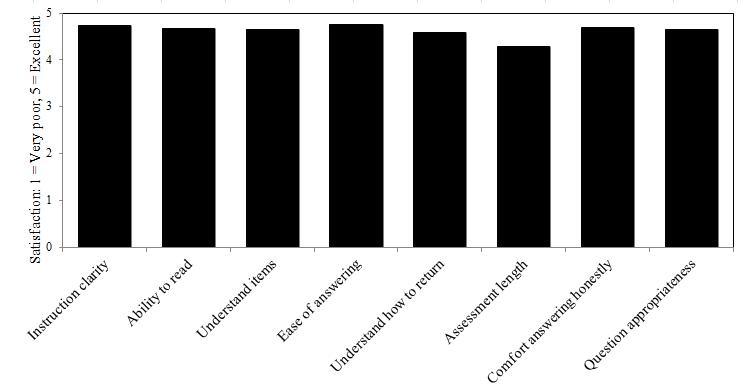
Mean CABIT assessment satisfaction scores for patients (n = 67). The target satisfaction score was 4.00 (Good).

**Figure 8:**
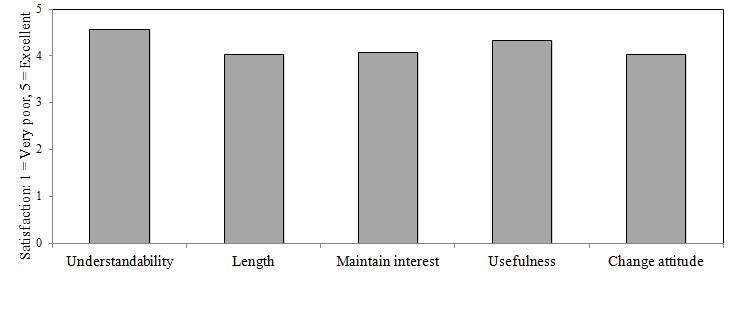
Mean CABIT video satisfaction scores for patients (n = 67). The target satisfaction score was 4.00 (Good).

**Figure 9:**
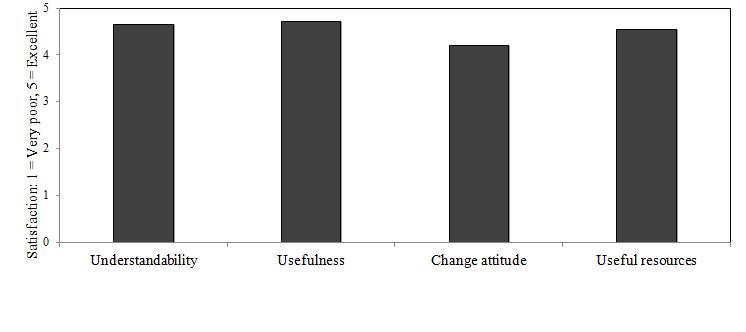
Mean CABIT Patient Tobacco Feedback Report satisfaction scores (n = 67). The target satisfaction score was 4.00 (Good).

Satisfaction ratings on all categories for the assessment, video intervention, and patient feedback reports were above our goal of a mean ≥ 4.00 (Good), (*M* = 4.48; *SD* = 0.70). Figures 7-9 illustrate domain satisfaction scores for participants. The items of relative weakness were the length of the assessment, length of the videos, interest of the videos, and the potential for the videos to motivate change. Qualitative evaluations also reinforced that the length of the assessment, as well as the ability of the videos to engage and motivate, while acceptable, could be improved. Suggested improvements for the videos included making the narrator more interesting and matched to the end user, presenting personal testimonials, and culturally tailoring the content by addressing issues that are of particular concern for different racial or ethnic groups.

##### Satisfaction Assessment with Depth Interviews

Themes that arose from the 15 depth interviews (5 based on each component: assessment, video intervention, and tailored patient feedback report) were integrated into the Master Theme Summary. Themes included in the Master Theme Summary were those endorsed by at least 3 respondents. Themes for the assessment included: the questions were understandable; instructions were clear; it was clear how to navigate the screens; the assessment length was appropriate; and it would be practical to administer this during visits to an emergency department, tobacco dependence program, or employee assistance program. Regarding the educational videos, the themes included: the video had good information; the video was not as useful as it could be; the situations portrayed in the videos are universal to all smokers; the videos were the appropriate length to hold your interest; the video format was useful; and the video was easy to understand. For the tailored feedback reports, the themes included: the report was informative; nothing should be changed with the report; the report was useful; the report was tailored to the participant; the report was the appropriate length and well-formatted; the report was understandable; and the report increased motivation to quit.

#### Health Care Provider Satisfaction

Of the 43 participants who completed the CABIT in the Emergency Department or Tobacco Dependence Program, 39 (91%) had a physician, nurse, or counselor complete satisfaction ratings of the Health Care Provider Report. Mean ratings exceeded our goal of 4.00 (Good) across all domains (*M* = 4.31, *SD* = 0.62), including: understandability (*M* = 4.44, *SD* = 0.55), usefulness (*M* = 4.26, *SD* = 0.68), length (*M* = 4.23, *SD* = 0.67), and overall format (*M* = 4.31, *SD* = 0.57). Providers indicated that the assessment gave them information not gathered during their standard evaluation for 34 out of 39 patients (87%). Additionally, 35 out of 39 patients (90%) received a referral that would otherwise not have been provided during routine clinical care.

### Completion Time

The median completion time was 22 minutes (IQR: 14-26 minutes). This time for the assessment alone included interruptions by providers, which were impossible to quantify, so the recorded completion times overestimate the true administration time by an unknown amount. The educational videos, which were 6-7 minutes in length, were not included in the time it took to complete the assessment.

### Dynamic Referral

Of the 45 participants not currently in treatment who expressed interest in changing their tobacco use and were offered a dynamic referral, 28 (62%) agreed to have their information sent to a best-matched tobacco treatment provider. While we did not assess the reason for not accepting a dynamic referral, we suspect these patients were not ready to quit or not interested in getting assistance to quit.

### Treatment Initiation

Of the 64 participants who were followed (ie, Emergency Department and Employee Assistance Program participants), we successfully contacted 44 (69%) for the follow-up assessment. We did not follow-up with patients from the Tobacco Dependence Program because they were already enrolled in treatment. Based on the follow-up information from the 44 participants contacted, combined with follow-up data obtained from the tobacco treatment sites where dynamic referrals were sent, we determined that 6 out of 64 patients (9%) had initiated tobacco treatment within 8 weeks of their baseline assessment. Of these 6 patients, 5 had received a dynamic referral and 1 had received a passive printed referral at the completion of the CABIT. Reasons for not entering treatment included not being ready, disliking the programs, living too far away, other appointments, transportation problems, and medical problems or surgery.

### Tobacco Use at Follow-up

Of the 44 participants interviewed for the follow-up assessment, 21 (48%) reported going at least 24 hours without smoking even a puff in the past 4 weeks (ie, a quit attempt), and 4 (9%) reported abstaining from tobacco use in the 7 days prior to the follow-up phone call (ie, 7-day point prevalence abstinence).

## Discussion

While research supports the effectiveness of provider-based interventions for improving tobacco cessation [3,48], clinicians often lack the time, training, and resources to carry out these interventions [1,2]. The CABIT program was created to help overcome these barriers by providing a brief individualized intervention with feedback in “real time,” a stage-matched video intervention, and optional dynamic referral to a tobacco cessation provider. Additionally, the CABIT required little staff time because it is self-administered, making it easier to integrate into busy medical settings.

In the Emergency Department, of the 426 patients approached to be screened for the study, and of those who were eligible, 43 were enrolled in the study. Twenty-four participants responded to emails to participate in a tobacco treatment intervention through the Employee Assistance Program and 3 patients who were in a Tobacco Dependence Program agreed to participate in the study after being asked by counselors. Three patients failed to complete the program in the Emergency Department because they were discharged or sent for testing or a procedure. Overall, there were 67 participants who completed the CABIT program.

Clinicians found the program to be useful. They rated the understandability, length, usefulness, and overall format of the Health Care Provider Report between 4 (Good) and 5 (Excellent) on a 5-point scale. Additionally, the CABIT proved to be useful to clinicians by providing information not obtained in the standard clinical assessment for 34 of 39 patients (87%). Providers indicated that 35 out of 39 patients (90%) evaluated would not have received a referral to a tobacco cessation program if the CABIT had not been administered.

For automated interventions to be widely disseminated into clinical practice in medical settings, they will need to be brief so they do not impede clinical flow. Our early end-user input from a range of health care providers suggested that the entire intervention, from start to finish, should be completed within 10 minutes for the majority of patients. The CABIT fell short of this goal, with a mean time of 22 minutes. It is important to note that this estimate is contaminated by down-time arising from interruptions from health care providers, especially in the Emergency Department setting. Additionally, we were overly inclusive in our assessment instruments, which included considerable redundancy. Eliminating the redundancy would undoubtedly shorten the assessment. Additional work will have to be done on the CABIT program to shorten the length of administration before efficacy testing can be completed. In developing computerized clinical interventions, a careful balance must be struck between obtaining enough information to be useful to individuals using the system and the strong demands to have a simple, efficient system that does not impede clinical flow. However, despite the shortcomings, the result of only 3 out of 43 patients in the Emergency Department failing to complete the assessment due to discharge or clinical care supports the feasibility of a program like the CABIT and the willingness of patients to participate even in a fast-paced environment.

Participants rated all aspects of the CABIT assessment, stage-matched video intervention, Patient Tobacco Feedback Report, and treatment referral locations between 4 (Good) and 5 (Excellent) on a 5-point scale. Connecting individuals with specialized tobacco treatment is an important goal of the CABIT program, considering the evidence that smokers who quit with assistance are more likely to succeed [3]. The dynamic referral proved to be a highly attractive component of the CABIT program with 28 of the 45 current tobacco users (62%) who were interested in quitting accepting the referral offer. Six (9%) of the participants we followed after baseline enrollment initiated tobacco dependence treatment with a specialist. The significance of this is difficult to evaluate, since we did not include a control condition. However, a previous study conducted with 577 smokers treated in an emergency department found that < 1% initiated treatment after they received a passive referral [32]. While 9% may not seem large in absolute terms, it may represent a significant increase in treatment engagement compared to treatment as usual (a passive referral). Moreover, even small effect sizes can translate into important public health and economic benefits. For example, the United States Preventive Services Task Force (USPSTF) recommends that primary care providers universally screen for tobacco use and give brief counseling. This recommendation is based on fairly modest increases of about 3-5% in abstinence rates over control conditions [49]. Further randomized, controlled clinical studies should provide more of a definitive evaluation of whether dynamic faxed referrals can promote treatment initiation and, ultimately, abstinence when compared to brief advice alone or passive printed referrals.

### Limitations

Limitations to the study included sample selection bias, which may have been present for those who were illiterate or not able to read at an eighth-grade level and for those who were computer illiterate despite our effort to assure patients that no computer knowledge was needed. Additionally, sample selection bias may have been present for those we excluded due to reasons of being too sick, not speaking English, and concerns about cognitive limitation. The sample size was relatively small, though this is mitigated by the proof-of-concept nature of the study. Follow-up limitations were possible with the 4- to 8-week follow-up window, which may have been too brief to catch all patients initiating treatment. Lastly, patient tobacco cessation at follow-up was based on patient report and not validated through biochemical means. Since this was not an efficacy trial, tobacco cessation was a secondary analysis.

### Conclusion

The CABIT proved to be an innovative and usable program that assisted providers in identifying tobacco users, providing brief individualized treatment with the stage-matched video intervention and feedback reports, and providing an automated referral to a tobacco treatment specialist. The program was highly accepted, easily implemented, and elicited a high level of satisfaction. Phase II of the CABIT will address the creation of a more user-friendly program, including a shorter assessment and production of videos that are more engaging and motivational. Lastly, future clinical trial testing is warranted to assess efficacy in promoting treatment engagement and tobacco cessation.

## Multimedia Appendix 1

Example of a Patient Tobacco Feedback Report from the CABIT program.

## Multimedia Appendix 2

Example of a Health Care Provider Report from the CABIT program.

## Multimedia Appendix 3

Example of a Tobacco Treatment Referral from the CABIT program.

## References

[1] Berlin I (2008). Physician's perceived barriers to promoting smoking cessation. J of Smoking Cessation.

[2] Vogt F, Hall S, Marteau TM (2005). General practitioners' and family physicians' negative beliefs and attitudes towards discussing smoking cessation with patients: a systematic review. Addiction.

[3] Fiore MC, Jaen CR, Baker TB, Bailey WC, Benowitz NL, Curry SJ, Dorfman SF, Froelicher ES, Goldstein MG, Healton CG, Henderson PN, Heyman RB, Koh HK, Kottke TE, Lando HA, Mecklenburg RE, Mermelstein RJ, Mullen PD, Orleans CT, Robinson L, Stitzer ML, Tommasello AC, Villejo L, Wewers ME (2008). Treating tobacco use and dependence clinical practice guideline, update.

[4] Thorndike AN, Regan S, Rigotti NA (2007). The treatment of smoking by US physicians during ambulatory visits: 1994–2003. Am J Public Health.

[5] Boudreaux ED, Bedek KL, Gilles D, Baumann BM, Hollenberg S, Lord SA, Grissom G (2009). The Dynamic Assessment and Referral System for Substance Abuse (DARSSA): development, functionality, and end-user satisfaction. Drug Alcohol Depend.

[6] Cupertino AP, Richter K, Cox LS, Garrett S, Ramirez R, Mujica F, Ellerbeck EF (2010). Feasibility of a Spanish/English computerized decision aid to facilitate smoking cessation efforts in underserved communities. J Health Care Poor Underserved.

[7] Finkelstein J, Lapshin O, Cha E (2008). Feasibility of promoting smoking cessation among methadone users using multimedia computer-assisted education. J Med Internet Res.

[8] Hoffman AM, Redding CA, Goldberg D, Añel D, Prochaska JO, Meyer PM, Pandey D (2006). Computer expert systems for African-American smokers in physicians offices: a feasibility study. Prev Med.

[9] Portnoy DB, Scott-Sheldon LA, Johnson BT, Carey MP (2008). Computer-delivered interventions for health promotion and behavioral risk reduction: a meta-analysis of 75 randomized controlled trials, 1988-2007. Prev Med.

[10] Swartz LH, Noell JW, Schroeder SW, Ary DV (2006). A randomised control study of a fully automated internet based smoking cessation programme. Tob Control.

[11] Tsoh JY, Kohn MA, Gerbert B (2010). Promoting smoking cessation in pregnancy with Video Doctor plus provider cueing: a randomized trial. Acta Obstet Gynecol Scand.

[12] Unrod M, Smith M, Spring B, DePue J, Redd W, Winkel G (2007). Randomized controlled trial of a computer-based, tailored intervention to increase smoking cessation counseling by primary care physicians. J Gen Intern Med.

[13] McDaniel AM, Casper GR, Hutchison SK, Stratton RM (2005). Design and testing of an interactive smoking cessation intervention for inner-city women. Health Educ Res.

[14] McDaniel AM, Benson PL, Roesener GH, Martindale J (2005). An integrated computer-based system to support nicotine dependence treatment in primary care. Nicotine Tob Res.

[15] Boyle RG, Solberg LI, Fiore MC (2010). Electronic medical records to increase the clinical treatment of tobacco dependence: a systematic review. Am J Prev Med.

[16] Cantrell J, Shelley D (2009). Implementing a fax referral program for quitline smoking cessation services in urban health centers: a qualitative study. BMC Fam Pract.

[17] Koplan KE, Regan S, Goldszer RC, Schneider LI, Rigotti NA (2008). A computerized aid to support smoking cessation treatment for hospital patients. J Gen Intern Med.

[18] Linder JA, Rigotti NA, Schneider LI, Kelley JH, Brawarsky P, Haas JS (2009). An electronic health record-based intervention to improve tobacco treatment in primary care: a cluster-randomized controlled trial. Arch Intern Med.

[19] Prokhorov AV, Yost T, Mullin-Jones M, de Moor C, Ford KH, Marani S, Kilfoy BA, Hein JP, Hudmon KS, Emmons KM (2008). "Look at your health": outcomes associated with a computer-assisted smoking cessation counseling intervention for community college students. Addict Behav.

[20] Etter JF, Perneger TV (2001). Effectiveness of a computer-tailored smoking cessation program: a randomized trial. Arch Intern Med.

[21] Prochaska JO, Velicer WF, Fava JL, Rossi JS, Tsoh JY (2001). Evaluating a population-based recruitment approach and a stage-based expert system intervention for smoking cessation. Addict Behav.

[22] Revere D, Dunbar PJ (2001). Review of computer-generated outpatient health behavior interventions: clinical encounters "in absentia". J Am Med Inform Assoc.

[23] Miller WR, Rollnick S (2002). Motivational Interviewing, Second Edition: Preparing People for Change.

[24] Prochaska JO, DiClemente CC (1983). Stages and processes of self-change of smoking: toward an integrative model of change. J Consult Clin Psychol.

[25] Prochaska JO, Velicer WF (1997). The transtheoretical model of health behavior change. Am J Health Promot.

[26] Janis IL, Mann L, Greenwald A, Brook T, Ostrom T (2005). A conflict-theory approach to attitude change and decision making. Psychological Foundations of Attitudes.

[27] Janis IL, Mann L (1977). Decision making: a psychological analysis of conflict, choice, and commitment.

[28] Bandura A (1977). Social learning theory.

[29] Kahneman D, Tversky A (1979). Prospect Theory: An Analysis of Decision under Risk. Econometrica.

[30] Boudreaux ED, Baumann BM, Friedman K, Ziedonis DM (2005). Smoking stage of change and interest in an emergency department-based intervention. Acad Emerg Med.

[31] Boudreaux ED, Kim S, Hohrmann JL, Clark S, Camargo CA (2005). Interest in smoking cessation among emergency department patients. Health Psychol.

[32] Ozhathil DK, Abar B, Baumann BM, Camargo CA, Ziedonis D, Boudreaux ED (2011). The effect of removing cost as a barrier to treatment initiation with outpatient tobacco dependence clinics among emergency department patients. Acad Emerg Med.

[33] Centers for Disease Control and Prevention (CDC) (2006). Behavioral Risk Factor Surveillance System Survey Questionnaire.

[34] Heatherton TF, Kozlowski LT, Frecker RC, Fagerström KO (1991). The Fagerström Test for Nicotine Dependence: a revision of the Fagerström Tolerance Questionnaire. Br J Addict.

[35] Ebbert JO, Patten CA, Schroeder DR (2006). The Fagerström Test for Nicotine Dependence-Smokeless Tobacco (FTND-ST). Addict Behav.

[36] DiClemente CC, Prochaska JO, Fairhurst SK, Velicer WF, Velasquez MM, Rossi JS (1991). The process of smoking cessation: an analysis of precontemplation, contemplation, and preparation stages of change. J Consult Clin Psychol.

[37] Velicer WF, Fava JL, Prochaska JO, Abrams DB, Emmons KM, Pierce JP (1995). Distribution of smokers by stage in three representative samples. Prev Med.

[38] Biener L, Abrams DB (1991). The Contemplation Ladder: validation of a measure of readiness to consider smoking cessation. Health Psychol.

[39] McKee SA, O'Malley SS, Salovey P, Krishnan-Sarin S, Mazure CM (2005). Perceived risks and benefits of smoking cessation: gender-specific predictors of motivation and treatment outcome. Addict Behav.

[40] Curry S, Wagner EH, Grothaus LC (1990). Intrinsic and extrinsic motivation for smoking cessation. J Consult Clin Psychol.

[41] Piper ME, Piasecki TM, Federman EB, Bolt DM, Smith SS, Fiore MC, Baker TB (2004). A multiple motives approach to tobacco dependence: the Wisconsin Inventory of Smoking Dependence Motives (WISDM-68). J Consult Clin Psychol.

[42] Velicer WF, Diclemente CC, Rossi JS, Prochaska JO (1990). Relapse situations and self-efficacy: an integrative model. Addict Behav.

[43] Brandon TH, Baker TB (1991). Psychol Assessment; 3(3):.

[44] Velicer WF, DiClemente CC, Prochaska JO, Brandenburg N (1985). Decisional balance measure for assessing and predicting smoking status. J Pers Soc Psychol.

[45] Bock BC, Becker B, Monteiro R, Partridge R, Fisher S, Spencer J (2001). Physician intervention and patient risk perception among smokers with acute respiratory illness in the emergency department. Prev Med.

[46] Hampson SE, Andrews JA, Barckley M, Lichtenstein E, Lee ME (2000). Conscientiousness, perceived risk, and risk-reduction behaviors: a preliminary study. Health Psychol.

[47] Kroenke K, Spitzer RL, Williams JB (2003). The Patient Health Questionnaire-2: validity of a two-item depression screener. Med Care.

[48] Stead LF, Bergson G, Lancaster T (2008). Physician advice for smoking cessation. Cochrane Database Syst Rev.

[49] U.S. Preventive Services Task Force (2009). Counseling and interventions to prevent tobacco use and tobacco-caused disease in adults and pregnant women: U.S. Preventive Services Task Force reaffirmation recommendation statement. Ann Intern Med.

