# Supplementary material for: The Computer-Assisted Brief Intervention for Tobacco (CABIT) Program: A Pilot Study
Source: J Med Internet Res. 2012 Dec 3;14(6):e163. doi: 10.2196/jmir.2074 (PMC3799483; doi:10.2196/jmir.2074)
Supplement: Supplementary file 1 [file jmir_v14i6e163_app1.pdf]

Cooper University Hospital

Patient Tobacco Feedback Report

Patient: SMITH, JOHN

Date of Birth: 01/31/1970 (42 years old)

Assessment date: 05/28/2012

Location: CABIT Reviewer Site

Your Referral Summary

At your request, your information was sent to the location with a “*”. Someone should call you within 5 days to talk about treatment options. If no one calls, you should call the telephone number provided.

* Cooper University Hospital
Stop Smoking Clinic
Three Cooper Plaza
Camden, NJ 08103
856-757-7736

National Toll Free Smokers Quitline
1-800-QUIT-NOW
(1-800-784-8669)
Telephone counseling

American Cancer Society
1-800-ACS-2345
(1-800-227-2345)

Patient Tobacco Feedback Report

Patient: SMITH, JOHN **Date of Birth:** 01/31/1970 (42 years old)

Assessment date: 05/28/2012 **Location:** CABIT Reviewer Site

The following information is based on your responses to the computerized assessment.

Report Outline

Your report is divided into the following sections:

A. Your Tobacco History
B. Your Readiness to Change
C. The Benefits of Quitting
D. Your Barriers to Change
E. Information on Resources

A. Your Tobacco History

You reported that you have used cigarettes and cigars and that you currently prefer cigarettes. You also indicated that you currently smoke 11-20 cigarettes a day and have been smoking cigarettes for about 27 years. Additionally, you noted that you have tried to quit in the past 12 months and that the longest you have ever been able to quit was for 1-7 days. This is great that you have had quit attempts in the past and shows you are motivated to try and quit.

B. Your Readiness to Change

Your Stage of Change

Based on the information you provided you are in the Contemplation Stage of Change. This means that you are thinking about quitting in the next six months and may even have attempted to quit in the past year. That's great news! You may not be ready to quit right now but you have been thinking about it. It's okay that you don't want to quit right now. You probably see both the good things and the bad things about your tobacco use. Most people who are thinking about changing, like you, find it be useful to think hard about the reasons they continue to use tobacco. What do you still get out of using tobacco? What are the things you are worried might happen if you quit? What is really more important to you – your tobacco use or your health? Many people think about quitting for a while before they actually attempt to quit. The decision to quit using tobacco is one of the most important health choices you can make. The benefits of quitting usually start immediately and continue for years after you have stopped. The following information will provide some helpful suggestions and help you to figure out some other things you can do to prepare yourself to quit using tobacco when you are ready.

Your Readiness Rulers

You answered several questions about your motivation to quit including the importance, readiness, and confidence in your ability to quit. Below you will find how you rated each item.

Importance of quitting (Your rating: 6)

1	2	3	4	5	**(6)**	7

Not at all important 100% important

Readiness to quit (Your rating: 7)

1	2	3	4	5	6	**(7)**

Not at all ready 100% ready

Confidence to quit (Your rating: 5)

1	2	3	4	**(5)**	6	7

Not at all confident 100% confident

In looking at your ratings, think about the reasons behind the scores you chose. Why did you rate each item as you did and not a lower score? What will it take to make each item a 7?

C. The Benefits of Quitting

How Quitting Benefits Your Health

There are many benefits to quitting tobacco use. The benefits that you indicated are: decreased chances of getting a serious illness, feeling better in general, feeling proud of yourself that you are able to quit, having more money and smelling better.

As you probably know, the U.S. Surgeon General warns that smoking cigarettes is bad for your health and increases your chance of getting many serious diseases. The most common are heart disease, stroke, cancer, and breathing problems like emphysema or bronchitis. However, did you know that your tobacco use makes you more likely to get the flu, pneumonia, bronchitis, and sleep and dental problems? Smoking also places you at risk for stroke, ulcers, and acid reflux.

You moderately agree that you currently have an illness that is caused or made worse by your tobacco use and slightly agree that your current healthcare visit is related to a problem stemming from your tobacco use.

You indicated that you believe your tobacco use will seriously damage your health and this is a possibility. However, the good news is that if you quit now, you can decrease your chances of damaging your health and even eliminate some of the potential problems.

Based on how much tobacco you have used in the past, you have already taken 3 years off your life. Think about it. Think about what you can do with the 11 minutes you take off your life each time you smoke a cigarette. And when you finish a pack of cigarettes, think about what you could have done with the 4 hours you just took off of your life. The good news is that if you quit now, you will decrease the chances of ending your life early from a smoking related illness. In fact if you quit now there is a good chance you will add 9 years to your life by preventing smoking related illnesses.

You will probably notice that once you quit smoking you will feel better. For example you may notice right away that food tastes better, your sense of smell is better, your clothing does not smell, your fingers and teeth will stop yellowing, and you will be able

to breathe better. In fact:

· **20 minutes** after you quit, your blood pressure drops to a level close to where it was before you had your last cigarette and the temperature from your hands and feet returns to normal.
· **8 hours** after you quit**,** the carbon monoxide level in your blood returns to normal.
· **24 hours** after you quit, you decrease your chances of having a heart attack.
· **1 to 9 months** after you quit, coughing, sinus congestion, fatigue and shortness of breathe decrease.
· **1 year** after you quit the excess risk associated with coronary heart disease decreases.
· **5 years** after you quit, your risk of having a stroke is reduced.
· **10 years** after you quit, your risk of dying from lung cancer is half of that of a continued smoker and you decrease your risk of mouth, throat, esophagus, bladder, kidney, and pancreas decreases.
· **15 years** after you quit, the risk of coronary heart disease is similar to someone who does not smoke.

How Quitting Benefits Others

Not only is cigarette smoke harmful to you, but also it is harmful to others around you, especially those who live with you who are exposed to the second hand smoke. You are placing others at risk for heart disease, cancer, stroke, clogging of their arteries, respiratory track infections, asthma, and COPD. The next time you light that cigarette around a loved one, think of how you are endangering their life.

How Quitting Benefits You Financially

Smoking probably costs you a lot of money. In fact, the price of cigarettes is likely to continue to rise. Based on the average costs of a pack of cigarettes per year in the U.S., since you started smoking, you have spent about $11,593.62. Think of what you could have done with that money such as pay rent, taken a vacation, bought a car, bought a house, paid for college tuition, or even bought a computer. The good news is that if you quit now based on your current rate of smoking and the current average price per pack ($6.00), you can save at least $135.00 per month. In one year you could save up to $1,642.50. Imagine what you could do with $1,642.50 in your pocket.

D. Your Barriers to Change

Your Level of Nicotine Addiction

As you may know, nicotine is what makes tobacco so addictive. Based on your responses, you are likely to have a moderate level of nicotine addiction. This is good news! Although quitting is almost always a hard thing to do, you may be more successful at quitting than if you were more strongly addicted. People who are not strongly addicted generally find that they do not have as many symptoms if they try to quit, like jitteriness, irritability, and difficulty concentrating. Even people who are not strongly addicted often say that quitting helps them to feel more in control of their lives. Even though you are not strongly addicted to nicotine, you may benefit from new treatments that can help people to quit smoking when you are ready. Keep in mind that this does not mean you are not at risk for smoking related illnesses. The only way to protect yourself from smoking related illnesses is to not smoke at all.

Your Temptations to Use Tobacco

Quitting is difficult and there are certain situations in which you may really crave tobacco. Here are some tips to manage the specific triggers you indicated were problems for you:

When you first get up in the morning: Morning is often a particularly difficult time for tobacco users, especially smokers. Sleeping is probably the longest time period your body goes without tobacco and therefore, the cravings in the morning can be intense. Many find giving up that first use of tobacco to be difficult especially when it is used with coffee in the morning. Coffee may not taste the same once you quit, too. That first tobacco use of the day sets the tone for the rest of day which usually involves finding the time for the next tobacco use. Some things you can do to help you in the morning would be to change your routine. Tobacco use is a habit and one thing you can do is change your routine to something that does not involve tobacco use in the morning. Start every day with some deep breathing and a healthy glass of water or juice. Make a list of some of the other triggers and how you can avoid them. Plan your morning so that you will be busy for that a period of time after you wake up. If you keep yourself busy, it will help you to not think about tobacco use. Place this trigger list or plan next to your bed so you are prepared when you wake up. Getting past the first tobacco use of the day is a big hurdle to overcome, but if you can get past this, it will make the rest of the day easier.

When you are anxious, angry, frustrated, or stressed: Many smokers feel the need for tobacco when they are feeling stressed. This is normal. But there are other things you can do to cope with stress instead of using tobacco. Make a list of some things you can do to help yourself relax such as thinking positive thoughts, listening to a relaxation tape or some of your favorite music, practice deep breathing (take a long deep breath and release slowly), take a walk, exercise, play with a pet, meditate, read, or call a friend. Prepare a list for when you are feeling anxious or stressed so that when you start to notice you are feeling that way, you are prepared.

Over coffee while talking and relaxing: Many people especially those who use cigarettes use tobacco while enjoying coffee or tea and relaxing. You can still do these things without tobacco. You should expect that you will have a strong urge to use tobacco since these two events have been “chained” to each other. One thing to remember is that your taste will become better with quitting tobacco use leading to the coffee and tea tasting different. Be very aware while you are having your coffee or tea. Take deep breaths between sip and breathe out slowly. Savor the taste and smell. Another tip that may help is to switch to decaffeinated. When people quit using tobacco, they can become irritable and nervous. This may be enhanced with caffeine. Keep you hands busy by having healthy snacks while drinking coffee or tea. Also to keep you hands busy, you can draw or make your plans for the day. Try to change up your plan and go out to get coffee or tea. Drink it in a restaurant where there is no smoking. If you find the urge hard to overcome, try drinking faster and engaging in other activities that you normally wouldn’t do while having coffee or tea to keep your body and mind busy. Some people are saddened that this special relaxation time is being taken away from them. Try to think of it from a different perspective by thinking of how quitting will benefit you.

When you are around others who are using tobacco: This is a temptation many tobacco users have. You see someone else using tobacco and it may trigger you to want tobacco. This trigger may even be that you just see the people who you once used tobacco with. Some ways to overcome this trigger would be to ask others to help in your attempt to quit by not using tobacco around you. If you would buy tobacco for others in the past, let them know that you can no longer do this for them. Let others know that you cannot hold their tobacco as well. If others around you start to use tobacco, remove yourself from the situation until they are finished. You can also do things such as placing a “No smoking” or “No tobacco use” sign on your door or in the window of your house and in your car if others often use tobacco in these places. Lastly, don’t let it bother you if anyone gives you a hard time about quitting. Just remind yourself about the benefits of quitting.

When you feel you need a lift or boost: Many tobacco users feel that tobacco provides them with a boost during the day. There are other ways to give yourself a boost during the day. Some people may associate this boost with taking a break from doing whatever they are doing. You can still take a break just do it without the tobacco. Just make a list of some things you can do and keep it handy so that when you feel you need a boost, you have a plan of action. Some activities could be exercising, taking a walk, getting a drink of water, or grabbing a snack. Try to get yourself up and moving.

When you realized you haven't had tobacco in a while: Many people feel the need for tobacco when they realize they haven't had any in a while. This shows how controlling tobacco can be. You can take control by keeping yourself busy. Try telling yourself that you are going to wait another hour before you have a cigarette. Keep yourself busy during that time. When the hour is up, if you still feel the need to have a cigarette, try to put it off a little longer. Remember the urges will pass. The goal is to learn how to "surf" them. Keep a list of things you can do to distract yourself to keep busy.

Your Perceived Risks of Quitting

As we mentioned quitting is difficult and sometimes the risks associated with quitting make it a less attractive alternative. However, there are ways to address these concerns. Some of the concerns you had were:

Miss the pleasure I get from tobacco: When you think about the pleasure you get from your tobacco use, ask yourself if it is worth your life. Sure, you may enjoy the tobacco but is that a strong enough argument for you to use it. Think of some other activities you enjoy doing. Think of some things that quitting tobacco use will help you to do.

Strong urges or cravings: Tobacco users usually typically have a certain level of nicotine in their body depending on the amount of use and type of tobacco used. When you don't have tobacco for a certain period of time such as when you are trying to quit, your body will want more nicotine, leading to cravings. When you experience triggers, or have a small amount of nicotine, your mood can change, your heart rate increases, and your craving increases. There is a physical and psychological component to cravings. When you quit, the craving will usually begin within one day depending on how much tobacco you use and what type. For example, smokers can expect to have cravings a couple hours after their last cigarette, and peak for a few days. These cravings may last for a few weeks, but keep in mind they will go away. These cravings will come and go and will be the strongest in the first week. The important thing to remember is to surf the urges. Expect that they will be there and plan how to handle them. Keep yourself busy when they come. They will only last a short amount of time. On occasion, you may experience repeated cravings right after each other. Just keep yourself busy and know they will go away. Try snacking on something healthy such as carrot sticks, celery, gum, or hard candy. Keep your mouth busy to help stop some of the psychological urges to smoke. Make sure to keep healthy snacks with you at all times because cravings can come at any time of the day. When you have an urge also try to take a deep breath in and out slowly through your nose. Try this a few times. Focus on your breath to help distract yourself from the urge. Most importantly, avoid high-risk situations when you quit until you feel confident to handle them. Stay away from those who are smoking and think of other specific triggers you have and how you can avoid them while you are quitting. Avoiding these situations now does not mean you must avoid them forever, just until you feel confident enough and you are far enough along in your quit attempt to handle these situations without using tobacco. Remember, the craving will pass. Give yourself a reward at the end of the day to help keep yourself motivated.

Feel like I'm losing a good friend: Many tobacco users feel like they are losing a good friend. In a sense you are. You may have used tobacco for a very long time. It has become a part of your life. It is there through the good times and the bad times, at any time of the day or night. It doesn't argue with you and will always listen. It is just like a good friend. Try thinking about it in a different way. If tobacco was a true friend, would it be slowly killing you and making you sick? Your true friends are those that will support you and help you through the quitting process. They will encourage you to quit and to make your health better. Make sure to utilize your friends and family in helping you through this time.

Less able to concentrate: Many smokers believe they will be less able to concentrate if they quit smoking. There are healthier alternatives to increase your concentration. First, pinpoint why you are getting distracted. Is the activity boring? Do you have too many things to do? Try finding ways to make the activity more interesting. You can break the activity into smaller tasks and switch tasks at certain time intervals when you find you are getting bored. Take frequent brief breaks such as taking a walk, or listening to music. Reward yourself when you get through a day without using tobacco.

Trouble dealing with boredom: Many tobacco users are concerned that it will be difficult to deal with situations when they are bored. For example, if you use tobacco as a break from work, you may find that when you take your breaks, you are bored. Other situations where you may feel bored include driving, or waiting for something or someone. This is normal. To help with these situations, try to keep yourself busy by eating healthy snacks to keep your hand and mouth busy, try bringing something to do during your breaks or if you need to wait, such as a magazine, a book, crossword puzzle, a game, or even a deck of cards. If you are waiting for someone or something, keep yourself moving and try not to stay in the same place for too long. Pay attention to what is going on around you such as the scenery or the people walking by. Use your senses to notice sounds, smells, feelings, and how things look. When you are driving, try chewing gum when you are in your car, find some good music on the radio that you can sing along to distract yourself.

Avoid a lot of people, places and activities: Many tobacco users are concerned that they will have to avoid a lot of people, places, and activities. Initially when you quit, you should try to avoid anything associated with tobacco use. This doesn't mean that you can't engage in activities you enjoy or be around people you enjoy. Eventually, when you are feeling confident in your ability to not use tobacco, you can resume these activities. Until then, stay away from places where people use tobacco and activities that often involve tobacco use such as when you are drinking alcohol. As for your friends, some may not be supportive of your quitting, particularly if they are tobacco users themselves. They may also not understand how their behaviors impact your efforts to quit. They may hassle you about quitting and make you feel that this will cause problems in your relationship. Don't let this influence you to start using tobacco. Try to stay around people that don't use tobacco or ask your friends if they could not use tobacco around you. If people around you start to use tobacco, excuse yourself. Your desire to use tobacco in these situations may not only be related to being around others who use tobacco, but also the particular situation such as taking breaks with people to use tobacco. What you can do is ask others to help you in quitting by asking them not to use tobacco around you, or ask you to purchase tobacco, hold their tobacco, lighter, or other tobacco related things. If others smoke or use tobacco outside of you home or in your car, post a "no smoking" or "no tobacco use" sign. Try not to get angry with others if they hassle you about quitting. You know the benefits and reason for your wanting to quit. Keep that in mind when you are faced with these struggles.

E. Information You Requested on Resources

Information About Treatment Options

Counseling or Medications from My Doctor

Your doctor can be helpful in your attempt to quit. They can provide you with information on nicotine replacement techniques and prescribe you medications to help deal with the withdrawal symptoms often associated with quitting. Speak to your doctor to get more information.

Counseling

Group and individual counseling can be very helpful in assisting you to quit or stay quit. Local programs are available to assist in your quitting by helping you deal with the urges and providing support and encouragement through your journey to quitting. Telephone counseling is also available as an option if you are unable to travel for counseling. The more intense the counseling the more likely you will be able to successfully quit. In the Referral Summary section at the beginning of this report, you will find information on local resources that provide these services.
