# Supplementary material for: The Computer-Assisted Brief Intervention for Tobacco (CABIT) Program: A Pilot Study
Source: J Med Internet Res. 2012 Dec 3;14(6):e163. doi: 10.2196/jmir.2074 (PMC3799483; doi:10.2196/jmir.2074)
Supplement: Supplementary file 2 [file jmir_v14i6e163_app2.pdf]

Cooper University Hospital

Tobacco Use: Healthcare Provider Report

****This report should be placed in the patient's medical record****

**Patient:** SMITH, JOHN	**Date of Birth:** 01/31/1970 (42 years old)
**Assessment date:** 05/28/2012	**Location:** CABIT Reviewer Site

This is a summary of a confidential assessment for the patient named above. It is designed to assist healthcare providers in providing tobacco counseling. Please review the information with the patient and sign in the space provided at the bottom of the report. The patient also should be given his/her Patient Feedback Report, which includes treatment resources for tobacco users.

Ask/Assess

Lifetime tobacco use:	Yes
Current use/Amount:	11-20 cigarettes / day
Years used:	27
Tobacco-related illness/symptoms:	chest pain, high blood pressure, asthma, acid reflux or heartburn, upper respiratory infection or cold, coughing in the morning, sleep problems
Methods used to quit in past:	cold turkey (quit without help), nicotine gum
Readiness to quit:	Ready to quit in the next 6 months (Contemplator)
Interested in counseling or medication from healthcare provider:	Yes

Healthcare Provider Should **Advise/Assist**

1. Inform the patient that quitting tobacco is one of the most important things he or she can do to improve his or her health.
2. Elicit change talk by having the patient describe the positive consequences anticipated if he or she quits.
3. At the patient's request, the patient's report also provides information about: group / individual tobacco cessation treatment

Refer/Arrange

· A tailored motivational report was printed for the patient.
· The patient viewed a stage-based video intervention.
· A faxed referral was sent to the referral source indicated with an "*".

* Cooper University Hospital<br>856-757-7736	National Toll Free Smokers Quitline<br>1-800-QUIT-NOW<br>(1-800-784-8669)	American Cancer Society<br>1-800-ACS-2345 (1-800-227-2345)

Healthcare Provider signature: ______________________ Date: ____________

☐ I reviewed and agree with the above assessment.

Provided counseling for:

☐ 0-3 minutes ☐ 3-10 minutes ☐ More than 10 minutes ☐ Did not counsel

Assessment Administrator Signature: ______________________ Date: ____________

This report reflects only the information supplied by the patient and is not intended to replace clinical judgment. The physician retains full responsibility for decisions regarding treatment. © 2008 Polaris Health Directions, all rights reserved. Contact Polaris at: (267) 583-6336 - info@polarishealth.com - www.polarishealth.com
