# Supplementary material for: The Computer-Assisted Brief Intervention for Tobacco (CABIT) Program: A Pilot Study
Source: J Med Internet Res. 2012 Dec 3;14(6):e163. doi: 10.2196/jmir.2074 (PMC3799483; doi:10.2196/jmir.2074)
Supplement: Supplementary file 3 [file jmir_v14i6e163_app3.pdf]

Cooper University Hospital
One Cooper Plaza
Camden, New Jersey 08103
Telephone: 856-757-7736
Fax: 856-757-9651

Fax Cover Sheet

TO: Nelson Byrne, Ph.D.

ORGANIZATION: Cooper University Hospital

DATE: 05/28/2012

FROM: Cooper University Hospital
Behavioral Medicine
856-673-4254

NUMBER OF PAGES: (including cover) 2

CONFIDENTIAL PATIENT INFORMATION

This information is being sent to you following a patient assessment conducted at Cooper University Hospital in Camden, NJ.

A patient that appears appropriate for your program / services has requested treatment. A report summarizing the results of the patient's assessment and providing contact information is attached. Please contact this individual within

WARNING: Unauthorized interception or use of this fax could be a violation of Federal and State law. If you have received this information in error, please notify the sender immediately.

This fax may contain confidential information belonging to the sender and may be used only for the purpose for which it was requested or intended. You are responsible for securing any confidential information.

This fax may contain health care information. Permission to use or disclose this information has been granted either by law or the patient. Further use or disclosure without additional patient authorization or as otherwise permitted by law is

Cooper University Hospital

Tobacco Treatment Referral

Please contact the patient as soon as possible

To: Nelson Byrne, Ph.D. **Fax #:** 856-757-9651 **Date:** 05/28/2012

Referred From: CABIT Reviewer Site **Phone #:** 856-757-7736

The patient below requested a referral for treatment and was told that someone from your organization would call him within 5 days.

Patient Information

Patient Name: SMITH, JOHN

Date of Birth (age): 01/31/1970 (42)

Assessment Date: 05/28/2012

Primary Telephone #: (111) 111-1111

Alternate Phone #: (111) 111-1111

Best time to call: Doesn't Matter

Okay to leave message? Yes

Assessment Summary

Current use/Amount:	11-20 cigarettes / day
Years used:	27
Tobacco product(s) ever used:	cigarettes, cigars
Level of addiction:	Medium
Tobacco-related illness/symptoms:	chest pain, high blood pressure, asthma, acid reflux or heartburn, upper respiratory infection or cold, coughing in the morning, sleep problems
Longest quit attempt:	1-7 days
Methods used to quit in past:	"cold turkey", nicotine gum
Readiness to quit:	Ready to quit, but not soon
Factors that predict a poor prognosis:	Lives with someone who uses tobacco

Confidence to quit (Patient rating: 5)

1	2	3	4	**((5))**	6	7

Not at all confident 100% confident

This report reflects only the information supplied by the patient and is not intended to replace clinical judgment. The physician retains full responsibility for decisions regarding treatment. © 2008 Polaris Health Directions, all rights reserved. Contact Polaris at: (267) 583-6336 - info@polarishealth.com - www.polarishealth.com
